# Implementation of Endoscopic Submucosal Dissection for Early Colorectal Neoplasms in Sweden

**DOI:** 10.1155/2013/758202

**Published:** 2013-06-27

**Authors:** Henrik Thorlacius, Noryia Uedo, Ervin Toth

**Affiliations:** ^1^Department of Clinical Sciences, Section of Surgery, Malmö, Skåne University Hospital, Lund University, 205 02 Malmö, Sweden; ^2^Department of Gastrointestinal Oncology, Osaka Medical Center for Cancer and Cardiovascular Diseases, Osaka 537-8511, Japan; ^3^Department of Clinical Sciences, Section of Endoscopy, Malmö, Skåne University Hospital, Lund University, 205 02 Malmö, Sweden

## Abstract

*Objectives*. Endoscopic submucosal dissection (ESD) is an effective method for en bloc removal of large colorectal tumors in Japan, but this technique is not yet widely established in western countries. The purpose here was to report the experience of implementing colorectal ESD in Sweden. *Methods*. Twenty-nine patients with primarily nonmalignant and early colorectal neoplasms considered to be too difficult to remove en bloc with EMR underwent ESD. Five cases of invasive cancer underwent ESD due to high comorbidity excluding surgical intervention or as an unexpected finding. *Results*. The median age of the patients was 74 years. The median tumor size was 26 mm (range 11–89 mm). The median procedure time was 142 min (range 57–291 min). En bloc resection rate was 72% and the R0 resection rate was 69%. Two perforations occurred amounting to a perforation rate of 6.9%. Both patients with perforation could be managed conservatively. One bleeding occurred during ESD but no postoperative bleeding was observed. *Conclusion*. Our data confirms that ESD is an effective method for en bloc resection of large colorectal adenomas and early cancers. This study demonstrates that implementation of colorectal ESD is feasible in Sweden after proper training, careful patient selection, and standardization of the ESD procedure.

## 1. Introduction

Colorectal cancer is one of the most frequent malignancies in the world and is the third leading cause of cancer-related death in Sweden. Neoplastic polyps (adenoma) are considered to be precursors of cancers in the colon and rectum. Stalked polyps can easily be removed by use of snare polypectomy. Sessile and flat adenomas can be eliminated efficiently using endoscopic mucosal resection (EMR) [1]. EMR is a relatively widespread and standardized method in western countries, but one problem is that large (>2 cm) sessile and flat adenomas are difficult to remove in one piece (“en bloc”) with EMR, and the endoscopist is usually forced to eliminate such tumors in multiple pieces (“piecemeal resection”). One disadvantage with fragmented polyps is that piecemeal resection makes it difficult for the pathologist to determine depth of invasion, lymphovascular infiltration, and lateral spread of the tumors in order to ensure radical removal. Uncertain radicality after EMR is a common reason for recommending surgical resection. Another disadvantage with piecemeal resection is that the rate of tumor recurrence is higher than that after en bloc resection [2, 3]. In order to avoid these problems associated with EMR, a new method referred to as endoscopic submucosal dissection (ESD) was developed in the late 1990s in Japan. ESD was originally developed to achieve en bloc resection of superficial neoplasms in the stomach. ESD enables removal of colorectal tumors without any size restriction. The limitation of ESD is oncological and risk of concomitant tumor cell spread to lymph nodes must be considered carefully. The consensus is that malignant tumors radically resected with ESD with less than 1000 *μ*m submucosal invasion do not need further surgical treatment [4–6]. Numerous studies have shown that ESD reduces local recurrence after removal of large early colorectal neoplasms compared to EMR [7–9]. The disadvantage is that ESD is much more technically challenging than EMR and that the frequency of complications is higher with ESD than EMR [8, 10–13]. Even in Japan, colorectal ESD is considered to be a difficult method, and intestinal perforations range between 1 to 10% in experienced hands [12–14]. Indeed, colorectal ESD is very uncommon in western countries. Considering that the number of early colorectal neoplasms will increase in parallel to colorectal cancer screening enhanced demands to treat early colorectal neoplasms with optimal minimal invasive techniques will be a challenge in the west. There are no structured training programs in colorectal ESD in western countries, and the implementation of colorectal ESD is currently dependent on individuals getting exposure and training in colorectal ESD in Japan. There have been no reports on colorectal ESD from Scandinavian countries in the literature. To increase the understanding and possibilities as well as the difficulties of implementing colorectal ESD in Scandinavian countries, we here report our experience in establishing and performing ESD for early colorectal neoplasia in a high volume center in Sweden. 

## 2. Methods

### 2.1. Patients and Tumors

From January 2012 to March 2013, 29 patients with early colorectal neoplasms underwent ESD at the Department of Endoscopy at Skåne University Hospital in Malmö, Sweden. Patients were considered eligible for colorectal ESD if they had a colorectal lesion larger than 20 mm in diameter or a local recurrence after EMR with extensive fibrosis. In principal, only cases with low- or high-grade dysplasia were enrolled for colonic ESD. Tumors showing evidence of regions of hardness, irregular nodules, ulceration, or submucosal tumor-like marginal elevation suggestive of submucosal invasion more than 1000 *μ*m were avoided. Certain patients with early rectal cancer (T1 stadium) were included if they were not fit for surgical resection due to extensive comorbidity. Tumor locations were divided into cecum, ascending colon, transverse colon, descending colon, sigmoid colon, and rectum. Patients were not included if they had lesions in the anal canal. Macroscopic classification of colorectal neoplasms included sessile lesions, which were elevated with a wide base and laterally spreading tumors (LSTs), which were defined as lesions 10 mm in diameter with a low vertical axis extending laterally along the interior luminal wall. LST lesions were further divided into two subtypes based on endoscopic appearance: LST-G type with even or uneven nodules on the surface and LST-NG type with a smooth surface.

### 2.2. Colorectal ESD

**Figure 1:**
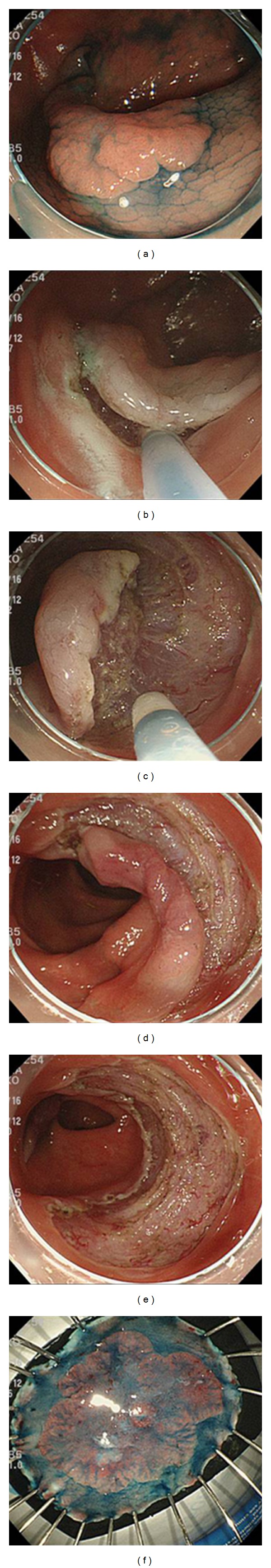
Standard procedure for colorectal ESD. (a) A large (3 × 4 cm) laterally spreading tumor-nongranular type in the transverse colon is delineated by use of topical application of indigo carmine. One can also see the frontal part of the disposable hood. (b) The lesion is elevated by submucosal injection of hyaluronic acid solution, and the anal part of the tumor has been incised by use of a Flush-knife. (c) The Flush-knife is used to dissect the submucosa and separate it from muscularis propria. (d) When approximately half of the lesion has been separated from the muscularis propria, the mucosal incision is completed around the lesion. (e) The lesion has been resected en bloc, and the remaining ulcer is examined for potential perforations and exposed blood vessels to coagulate. (f) The resected specimen is stretched and nailed to facilitate histological examination.

Colonic cleansing was based on intake of four liters of polyethylene glycol prior to the ESD procedure. All patients underwent conscious sedation by use of intravenous administration of midazolam hydrochloride (Panpharma, Fougères, France) and analgesia with ketobemidone chloride (Pfizer Inc. New York, USA). Midazolam administration was reiterated if necessary. An intravenous injection of 20 mg of scopolamine butylbromide (Buscopan, Boehringer Ingelheim, Ingelheim, Germany) or one mg of glucagon (Novo Nordisk A/S Bagsvaerd, Denmark) was given to reduce intestinal peristalsis. Carbon dioxide insufflation was used during colorectal ESD in order to minimize patient discomfort. A colorectal surgeon with extensive experience in invasive endoscopy (H.T.) performed all procedures. The main outcomes were en bloc and curative resection rate, procedural time, and complications. ESD procedures in the descending and sigmoid colon as well as the rectum were conducted with a gastroscope (GIF-H180J, Olympus, Hamburg, Germany) and in the cecum, ascending and transverse colon by use of a colonoscope (CF-H180AI, Olympus). Topical administration of 0.4% indigo carmine and narrowband imaging was used to delineate the lesions (Figure 1(a)). A disposable distal attachment (D-201-11804 or D-201-15004, Olympus) was mounted onto the tip of the endoscope. VIO 300D (ERBE Elektromedizin, Tübingen, Germany) was used as power source for electrical cutting and coagulation. To elevate the lesion, hyaluronate sodium solution (0.4%, Sigmavisc, Hyaltech Ltd, Livingston, UK) was injected into the submucosa using a 21-gauge injection needle (NM-400L-0421, Olympus) outside the tumor margin. Flush knife [15] with a 1.5 mm long tip (Fujifilm Europe GmbH, Düsseldorf, Germany) connected to a water jet pump was used to cut the mucosa (Figure 1(b)),  dissect the submucosa from muscularis propria (Figures 1(c) and 1(d)), and coagulate bleeding vessels. Submucosal injection of hyaluronate sodium (using the endoscopic needle) and water jet injection of saline solution by use of the Flush-knife reiterated during the procedure in order to maintain sufficient submucosa elevation during the procedure. A hemostatic forceps (Coagrasper, FD-411UR, Olympus) was used to stop larger bleedings or to prevent hemorrhage before vessel cutting. After removing the lesions, resected specimens were retrieved by use of grasping forceps (FG-47L-1; Olympus) or a basket (Roth Net, US Endoscopy, Mentor Ohio, USA). The post-ESD ulcer was carefully examined (Figure 1(e)), and pulsating vessel stumps were coagulated with the coagrasper using a soft Coagulation mode. Before completing the procedure, the margins of the ulcer were carefully investigated to ensure complete lesion removal (Figure 1(e)). The specimen was stretched and pinned onto a hard plate to facilitate histological examination (Figure 1(f)). Procedure time was defined as the time from incision with the Flush-knife to the completion of removal the lesion. 

### 2.3. Histological Evaluation

Resected specimens were immersed in 10% formalin and fixed specimens were sectioned serially at 2 mm intervals and subjected to histological examination. Vienna classification of gastrointestinal epithelial neoplasia was used to classify the colorectal neoplasms [16, 17]. En bloc resection was defined as resection in one piece of tissue. R0 resection was defined as tumor-free vertical and lateral margins. R1 resection was defined as incomplete resection with tumor cells extending into the vertical or lateral margins. Curative resection was defined as tumor-free vertical or lateral margins of the lesion and when submucosal invasion was not deeper than 1000 *μ*m without vascular or lymphatic involvement. 

### 2.4. Complications and Follow-Up

Perforation during an ESD procedure was classified into immediate and delayed perforations, during and after completion of the procedure, respectively. Perforations were defined as small holes with visible omentum or other tissue outside the muscle layer, such as transparent serosa, visualized endoscopically, and free air in the abdomen demonstrated on image studies. Procedure-related bleeding was defined as clinical evidence of hemorrhage with melena or hematochezia requiring a special hemostatic method after the ESD procedure. If a bleeding during the procedure caused abortion of the ESD intervention, it was considered to be a complication. Follow-up colonoscopy was planned 3–6 months after ESD. A biopsy was performed for histological assessment of any suspicious abnormality. 

### 2.5. Statistics and Ethics

Data are given as median and range. The ESD procedure was performed in accordance with the ethical principles of the Declaration of Helsinki. All patients received a detailed explanation of the procedure, including risks of bleeding, perforation, and the possibility of additional surgery due of complications or histological diagnosis of resected specimens. 

## 3. Results

### 3.1. Patients

**Figure 2:**
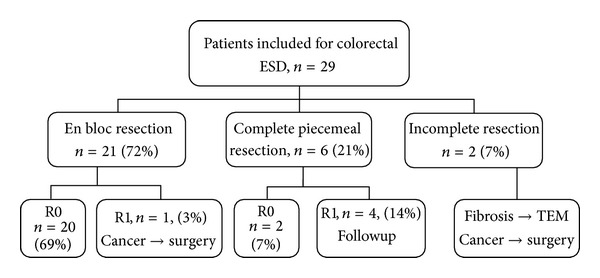
Outcome of colorectal ESD. ESD: endoscopic submucosal dissection; R0 : R0 resection; R1 : R1 resection; TEM: transanal endoscopic microsurgery.

Twenty-nine patients were included in the study of which 14 were males and 15 females (Figure 2). The median age of the patients was 74 years (range 46–85 years). Tumor size was 28 mm (range 11–89 mm). The smallest lesion measuring 11 mm was a case of local recurrence after previous EMR with extensive fibrosis. Preinterventional histology defined all tumors as adenomas with low- or high-grade dysplasia except for one rectal tumor with invasive cancer (invasive depth not determined), which could not undergo surgery due to advanced co-morbidity. Four tumors were located in the cecum (14%), two in the transverse colon (7%), and six in the sigmoid colon (21%) as well as 17 in the rectum (59%). The macroscopic types included 10 sessile (34%), seven LST-NG (24%), and 12 LST-G (41%) tumors (Table 1). 

### 3.2. ESD Performance

En bloc resection rate was achieved in 21 cases (72%), while R0 resection rate was achieved in 20 patients (69%) (Figure 2). Curative resection (en bloc + piecemeal resection) was obtained in 22 out of 29 patients (76%) (Figure 2). The median procedure time was 142 min (range 57–291). This sample is too small to identify factors related to a longer procedure time. Histological examination revealed low- or high-grade dysplasia in 24 patients (83%) (Table 1). Five patients were documented to have invasive cancer of which three had sm1 tumors in which ESD resulted in R0 resections and required no further interventions. Two patients had invasive cancers classified as more advanced than sm1 and consequently underwent surgical resection. One suspected immediate perforation occurred in a patient with a lesion in the cecum, which was closed with endoclips. One patient with a rectal tumor experienced post-ESD fever, and computer tomography showed suspected free gas outside the rectal wall. Both of these patients with suspected perforations could be managed conservatively with five days of diet restriction and antibiotics. Thus, two out of 29 patients had a perforation making the perforation rate in this study 6.9%. One case with a sigmoid tumor had a bleeding during the procedure, which was stopped by use of clips but led to abortion of the procedure. This patient had stopped ingestion of clopidogrel only five days prior to the intervention. No case with significant postoperative hemorrhage was noted. Also, all patients survived the ESD intervention. Five patients have undergone a follow-up endoscopy 3–6 months after ESD without detecting any local recurrence.

## 4. Discussion

The advantage of ESD compared to EMR is that large colorectal tumors can be removed en bloc, which facilitates the pathological evaluation of the specimen and reduces the risk of local recurrence compared to EMR. The disadvantage with ESD is that this method is technically challenging to learn and that the risk of perforation is higher than that of EMR. Although most ESD-associated perforations are treated conservatively and rarely require surgical intervention, some perforations are indeed fatal. These factors have restricted the dissemination of colorectal ESD in western countries. This present study shows that implementation of colorectal ESD is possible in Sweden and represents the first data on colorectal ESD performed at a specialized center in Scandinavia.

Piecemeal resection is associated with increased risk of incomplete resection and local recurrence [2, 3]. The local recurrence rate after piecemeal EMR of lesions larger than 20 mm ranges between 7 and 20% [3, 7, 8]. Numerous studies from Japan have convincingly shown that en bloc resection by use of ESD not only increases R0 resections but also reduces local recurrence rate down to 0–3% [8–10]. This study including 29 colorectal ESD cases during a period of less than two years conducted by one experienced surgical endoscopist under his initial learning curve detected an en bloc resection rate of 72% an R0 resection rate of 69%. These rates are lower than those reported by experienced centers in Japan where en bloc resection rate ranges between 84 and 99% [8–10, 12, 13] but in line with the limited experience reported in western centers ranging between 55 and 82% [18–23]. We observed no local recurrences although the followup time was not longer than 6 months in a limited number of patients. Further follow-up is required to obtain solid data on local recurrences in a longer perspective. The majority of the lesions in the colon and rectum were benign in this study (83%). Nonetheless, our material also included five cases with invasive cancer of which three were resected en bloc ESD showing submucosal invasion less than 1000 *μ*m without signs of lymphovascular engagement, tumor budding, or poor grading, suggesting that the risk of lymph node metastasis in these cases is less than 3% [4–6] avoiding the need of surgical intervention. These cases with R0 resection of superficial submucosal cancer are followed up endoscopically. In addition, our study contained two adenocarcinoma cases with submucosal invasion deeper than 1000 *μ*m, which underwent surgical resection. 

Intestinal perforation is the most feared complication in colorectal ESD. The rate of perforations varies between 1 and 10% in experienced centers in Japan [8–14]. Herein, we observed one perforation in the cecum and one in rectum leading to a perforation rate of 6.9%. Both these patients with perforation were conservatively treated with oral antibiotics for five days. A perforation rate of 6.9% is in line with reports from studies in Japan. However, it should be pointed out that the median size of the resected tumors in this study was 26 mm (range 11–89 mm) which is smaller than those reported from Japan [8, 10–13]. Knowing that the lesion size is an important factor related to the risk of peroration [24] and the fact that lesions with large ulcerations and substantial nonlifting signs were avoided in this study, it could be speculated that our perforation rate of only 6.9% could have been higher using other inclusion criteria. Nonetheless, we consider our perforation rate acceptable during a learning curve and in line with our studies conducted in western countries reporting perforation rates between 1 and 20% during colorectal ESD [18–23].

One challenge with ESD is that the procedure time is significantly longer than that of EMR. Herein, we observed that the median ESD procedure time was 142 min, which is not only longer than that of EMR but also significantly longer than procedural times (61–116 min) reported from experienced centers in Japan [7, 8, 13, 24]. This difference is not surprising knowing the large impact of case experience on ESD procedural times. For example, it has been reported that the procedural time was 200 min for the first third of the cases, which significantly decreased to 134 min in the last third of the cases in a series of 72 colorectal ESD cases [21].

One limitation of this study is that we did not perform a randomized trial comparing ESD and EMR. Such prospective randomized trials could be helpful to evaluate the role of ESD in the management of early and large colorectal neoplasms in Sweden. Our experience is that besides training in animal models, one should obtain direct experience from experts in Japan before attempting ESD in the gastrointestinal tract. It is also extremely helpful if experts from Japan can directly supervise when starting ESD programs outside Japan. In Japan, endoscopists start to do ESD in the stomach since the incidence of early gastric lesions is high there. In western countries where the frequency of early gastric lesion is low, one must rather start with ESD in the rectum. Once proficiency is obtained in doing rectal ESD, one can move forward and attempt ESD in the colon and esophagus. This model for learning ESD in Sweden is in line with recent European recommendations [25].

## 5. Conclusions 

We conclude that ESD is an effective method for en bloc resection of large adenomas and early cancers in the colon and rectum. Moreover, our results demonstrate that ESD is a safe method for managing large colorectal lesions when performed by an experienced interventional endoscopist combined with careful patient selection. Further studies are needed to compare ESD and EMR in terms of efficacy and cost benefit in randomized trials. Nonetheless, the present study suggests that implementation of colorectal ESD is feasible in Sweden.

## Figures and Tables

**Table 1:** Patient and tumor characteristics.

	Total
Total number of patients	29
Age (years)	74 (46–85)
Gender, *n*, (%)	
Male	14 (48%)
Female	15 (52%)
Tumor size (mm)	28 (11–89)
Tumor location, *n* (%)	
Cecum	4 (14%)
Transverse colon	2 (7%)
Sigmoid colon	6 (21%)
Rectum	17 (59%)
Macroscopic type, *n*, (%)	
Sessile	10 (34%)
LST-G	12 (41%)
LST-NG	7 (24%)
Histology, *n*, (%)	
Low-grade adenoma	19 (66%)
High-grade adenoma	5 (17%)
Adenocarcinoma, sm1	3 (10%)
Adenocarcinoma, >sm1	2 (7%)

LGT-G: laterally spreading tumor-granular type; LST-NG: laterally spreading tumor-nongranular type; sm1: submucosal invasion <1000 *μ*m; >sm1: submucosal invasion >1000 *μ*m.
